# "The Hospital" at One Penny

**Published:** 1911-09-23

**Authors:** 


### The Week's Work.

### "THE HOSPITAL" AT ONE PENNY.

On and after October 6 next The Hospital will be published at the popular price of ONE PENNY. While including, as hitherto, the expert treatment of all *hospital* subjects, institutional life in all its forms will be the subject-matter of the paper. The architect who designs the hospital, the secretary who organises, the surgeon, the patient, the dispenser, the almoner, the sanatoria staff, the members of the public services and all who take part in its labours will now have one official channel of news. The Hospital will thus become the official paper of all institutions dealing with every kind of human disablement, disease and accident, including all organisations charged with the public health and the physical well-being of the people. It should prove an indispensable friend of the profession, the institution, the family, and the individual, especially of those workers engaged in the medical, health, and preventive services everywhere. The complete scheme of the new paper will be published next week.

### HOW TO ATTEND THE MANCHESTER MEETINGS.

Some details concerning the arrangements made for members wishing to attend the Conference, on Thursday and Friday, September 28 and 29, of the British Hospitals Association, will be welcome. The following arrangements for travelling to Manchester have been made: The railway companies will issue tickets at reduced fares to members attending the Conference. Applications for vouchers for presentation at the booking offices must be made to Mr. W. G. Carnt, Hon. Local Secretary to the Conference, Royal Infirmary, Manchester. On arrival in the city hotel arrangements on the following scales have been made: The charges to members for bed and breakfast at the Midland Hotel will be 8s. to 12s., at the Queen's Hotel 7s. 6d., at the Grand Hotel 7s., at the Albion Hotel 6s. 6d., and at the Victoria Hotel 6s. On Thursday (or Saturday) afternoon, at 2.15, special electric cars will leave the Midland Hotel for the Pomona Dock, Old Trafford, whence visitors, upon the invitation of the Directors of the Manchester Ship Canal, will proceed by steamer to Barton Bridge. During at any rate one afternoon the following hospitals will be open for the inspection of members on presentation of the Conference card: Royal Infirmary, Royal Eye Hospital, St. Mary's New Hospital and the Dental Hospital in Oxford Road, Manchester; also the Northern Hospital for Women and Children, Cheetham Hill, Manchester; Hospital for Skin Diseases, Quay Street, Manchester; Salford Royal Hospital, Salford; Manchester Children's Hospital, Pendlebury; Ancoats Hospital, Mill Street, Ancoats. Tea will be provided for visitors.

### HOSPITAL CONFERENCES AT MANCHESTER AND NEW YORK.

The great interest which is being excited in the conference of the British Hospitals Association, which, as already announced, is to take place on Thursday and Friday, September 28 and 29, at Manchester, will inevitably draw the attention of all English hospital workers to a similar conference that is now in progress at New York. Last Tuesday the American Hospital Association opened its thirteenth annual conference. The Boards of Trustees of every hospital in Canada and the United States have been asked to send at least one delegate, and among the papers arranged for were the following: "The Sanitarium Hospital," by Dr. J. H. Kellogg, Battle Creek Sanitarium, Battle Creek, Mich. "Concerning European Hospitals," by Dr. J. N. E. Brown, Secretary, Toronto, Ont. "Hospital Treatment of Communicable Diseases," by Dr. D. L. Richardson, Providence City Hospital, Providence, R.I. "Some Problems in the Dietary Department of Hospitals," by Miss E. Grace McCullough, Dietitian, Mass. General Hospital, Boston, Mass. "The Development of Typhoid Fever among Hospital Workers," by Mr. J. M. Cosgrave, Manager Winnipeg General Hospital, Winnipeg, Man. Another interesting subject is "Hospital Annual Reports. What for? Who for?" by Mr. J. H. S. Parke, Secretary Montreal General Hospital, Montreal, Quebec. A paper on "The Foundation of Hospital Efficiency" will be read by Mr. Frank J. Firth, Germantown, Philadelphia, Pa. "Hospital Facilities in New York City" will be discussed by Mr. Robert W. Hebberd, Secretary State Board of Charities, Albany, New York. Other subjects include "Hospital Organisation in Relation to Education and Research," by Mr. Abraham Flexner, of the Carnegie Foundation, New York City. Hospital architects will be interested in the Report of Committee on Hospital Construction, by Mr. Edward F. Stevens, 9 Park Street, Boston, Mass., and hospital committeemen in the Report of Committee on Hospital Efficiency, Hospital Finances, and Economics of Administration, by the Rev. A. S. Kavanagh, Methodist Episcopal Hospital, Brooklyn, New York. An interesting feature also is the round-table conference for superintendents of small hospitals, of which the chairman is Miss Mary L. Keith, Rochester City Hospital, Rochester, New York. There is also to be a non-commercial exhibit of hospital appliances, invented, approved, or arranged by hospital workers, of which Miss Charlotte A. Aikens, the author of "Hospital Management," is to be general chairman.

### HOW TO JOIN THE BRITISH HOSPITALS ASSOCIATION.

While following attentively the conference in New York, we may claim that recent legislative proposals in this country offer a programme of subjects that can hardly have been equalled in interest by any handled at previous hospital conferences on either side of the Atlantic. If there be any hospital officers who have not yet become members of the British Hospitals Association we urge them to join in time for the Manchester conference, the attendance at which is certain to be very large, and should be altogether comprehensive. Mr. Conrad Thies, the honorary treasurer, is still ready to receive forms of application for membership, which, accompanied by a remittance of half-a-guinea, can be obtained from, and should be returned to, him at the Royal Free Hospital, London.

### THE KING AND CARDIFF INFIRMARY.

If strenuous efforts be the way to gain the Royal approval, then the news that has been just announced, that the King has been pleased to approve of the Cardiff Infirmary being styled the King Edward VII. Hospital, Cardiff, will come as no surprise. It should be no surprise anyway to readers of "The Hospital," for during the past year the story of the institution that was making probably the chief efforts at consolidating its future, and which has been very carefully followed in our pages, has been the story of the Cardiff Infirmary appeal. Mr. Leonard Rea, Colonel Bruce Vaughan, and several others, especially have distinguished themselves, and we very much hope that the board of management may succeed in securing the presence of his Majesty at the future opening ceremony. With so energetic a chairman as Colonel Bruce Vaughan we need not fear that the hint contained in Mr. Byrne's letter from the Home Office: "The board may then (next year), perhaps, submit the proposal (for the King to open the new wing) again to his Majesty's private secretary"—will be wasted on unappreciative ears. The progress made at Cardiff has been made under the inspiration of the voluntary system, and the voluntary system throughout the country would respond most enthusiastically to the royal approval of its maintenance which a visit from King George to the King Edward Hospital, Cardiff, would certainly confer.

### TUBERCULIN TREATMENT IN SHEFFIELD.

Through the generosity of Mr. Douglas Vickers, the Sheffield City Council will be enabled to give an extended trial to the tuberculin treatment of consumption. Among the cutlers of Sheffield there is, as is well known, a high proportion of sufferers with tuberculosis, and sanatoria have already been established by the City Council. Now a gift of £500 a year for two years has been placed at their disposal the authorities have appointed Dr. Chapman, formerly at the Infirmary, to superintend the treatment of patients with tuberculin.

### THE SPEAKERS AT THE MEDICAL SESSION.

Monday and Tuesday, October 2 and 3, have been chosen for the opening of the winter medical session at the majority of the London Hospital Medical Schools. At St. Mary's Hospital the opening will take place on Monday, October 2, when the introductory address will be delivered at 3 p.m. by Lord Justice Fletcher Moulton, who will also present the prizes for the past session. On the following night, Tuesday, October 3, the annual dinner of the past and present students will be held at Princes' at 7 p.m., when Mr. A. T. Norton, C.B., F.R.C.S., consulting surgeon to the hospital, will take the chair. Prince Alexander of Teck will preside at the opening of the Middlesex Hospital Medical School on October 2, when Dr. Comyns Berkeley will address the students. The prizes will be distributed by Mr. Marconi. The banquet, it should be observed, will be held the same evening at the Trocadero Restaurant. At Charing Cross Hospital Mr. Robin Duff, chairman of the council, will preside at the distribution of prizes. The London School of Medicine for Women has secured Sir Henry Butlin for an address on "Research in Medicine, and Women in Medical Research." At University College Hospital Sir William Ramsay will distribute the prizes. At St. George's Hospital Dr. Henry Miers, Principal of London University, will give the address. Finally, on October 3 at the London Hospital the first of two Schorstein Memorial Lectures will be delivered by Dr. James Mackenzie on "Auricular Fibrillation." It is somewhat late in the day, but at St. Thomas's Hospital no chairman has yet been selected for the prize distribution. At King's College Hospital there will be no prize distribution. It is a propagandist and social rather than an institutional or scientific interest which, as usual, is likely to attach itself to these functions. Comparison with the programmes of previous years is therefore not to be pressed too closely. Still we may note that a year ago the lecturers were, on the whole, more of that type of personality aimed at by the Romanes lectureships at Oxford than they can be said to be on this occasion. Lord Kitchener, for instance, is conspicuous by his absence, but it will strike the most casual observer that Sir William Ramsay's address at University College is likely to provoke the greatest amount of comment. We have little doubt he will make serious use of the attention he commands from all sections of the public.

### DOCTOR'S DEATH AT AN OPERATION.

The sudden death is reported of Dr. Alexander Gray, of Bradford. Last week he visited the operating theatre of the Bradford Royal Infirmary to witness an operation, when he was seized with an attack of angina pectoris and died in a few moments. Dr. Gray graduated at the University of Glasgow in 1878. He was a prominent practitioner in Bradford, and among other appointments he held that of divisional surgeon to the Bradford police.

### NEW RULES FOR APPOINTMENTS AT IPSWICH HOSPITAL.

The election of the members on the honorary staff of the East Suffolk and Ipswich Hospital last week has peculiar interest from the fact that it has taken place after an important revision of the rules. A vacancy which occurred on the honorary staff was filled, at a meeting of the Board of Management specially convened for that purpose, by the re-election of Mr. A. Y. Pringle, M.R.C.S., L.R.C.P., who was originally elected in 1901 for ten years, the present election being for a similar term. There were no other candidates for the office. At the same meeting the re-election of the assistant honorary staff of three members took place. All the old members sought re-election, as did two new candidates, who, however, did not prove sufficiently strong to oust those whose term of office was about to expire, and on a poll being taken it was found that the three old members—Mr. J. F. C. Hossack, L.R.C.S., F.R.C.S., Mr. C. K. Moseley, M.R.C.S., L.R.C.P., and Dr. W. A. Gibb, M.D., C.M.—were returned, the term of office being fixed at five years. As stated above, the rules of this hospital have recently been undergoing some revision, and no section has been altered more than that relating to the election of the honorary staff. Under the old order the election of all members of the staff was supposed to be undertaken by the Governors of the hospital—a governor being an annual subscriber of two guineas or a donor of £30 and upwards; in other words, the decision was left in the hands of an unwieldy body of over six hundred persons who took little real interest in the active management of the institution. This has now been altered to the full Board of Management, which consists of some ninety members. The above election is the first to take place under the new régime.

### A MODEL OF JUDICIOUS ADVERTISING.

The Manchester Children's Hospital at Pendlebury is now known as the most efficient of children's hospitals. This actual phrase indeed was used by an authority who went out of his way to place on record his belief that its efficiency when inspected by him nearly thirty years ago has been more than maintained in a recent visit. The latest pamphlet issued by the institution shows that it is not only the health of the children which is attended with happy results. This leaflet is indeed a capable piece of work: not exactly a history, not obviously an appeal, not transparently an illustrated photograph book of benefactors and subscribers, it combines the advantages of a judicious historical sketch, of an insidious appeal for money, with the pleasant attraction of good illustrations. Mr. Eason, the secretary—for we are sure that we can trace his experience and tact in this little publication—is to be congratulated sincerely on this recent example of hospital propaganda. It is characteristic that the northern hospital, probably in the least need of advertisement, should as this pamphlet shows, be advertised with the greatest amount of discrimination? Good management reacts all round.

### THE ANNUAL WASTE OF DRESSINGS.

It is stated in a Maltese paper that a patient at the local Government Dispensary was asked by one of the district medical officers to wash and to return the bandage with which his wound had been dressed. Economy, apparently, was the aim of this request. There is no doubt that many pounds a year are wasted in our voluntary hospitals by the destruction, careless or intentional, of hospital dressings. The Moabite Hospital in Berlin can teach a very valuable lesson to our students and ward-sisters in this respect. There all the dressings are sterilised, and though twice the number of in-patients are treated every year, the bill for dressings is only half that which is current in the largest of our voluntary hospitals.

### IMPORTANT EXTENSIONS AT GLASGOW.

We understand that the arrangements for the opening of the clinical laboratory and of the new wards now all but finished at the Western Infirmary, Glasgow, have been completed. Sir Mathew Arthur, Bart., as chairman of the Board of Management, will preside, and the date of opening has been fixed for Saturday, October 6. The opening ceremony will be undertaken by Sir James Barr, M.D., the president-elect of the British Medical Association.

### THE BEST TIME FOR ISSUING APPEALS.

Some months ago, as the result of certain recommendations by the King's Fund, an appeal was issued by the Samaritan Free Hospital for Women in Marylebone Road for £3,500 to provide iron staircases for use in case of fire, and also to rebuild the nurses' home. So far £1,200 has been raised. Mr. W. Guntrip King, the secretary, thinks the whole might have been raised before now had it not been for the holidays and the strikes. Perhaps so. In any case we are glad to remind the public that any need which has the backing of the King's Fund deserves most heartily the backing of all hospital supporters, and to remind Mr. King that a life-long experience of raising sums of money for the hospitals has taught us that the autumn is the time of year most suitable for the issue of an appeal. Just because the sum of money now wanted by the Samaritan Free is a small one, it must not be allowed to impede the hospital's work. We hope, therefore, October will see Mr. Guntrip King and his staff quick to take the opportunity that the autumn invariably affords for hospital appeals to be issued with success.

### THE RUSSIAN DOCTORS' DEBT TO M. STOLYPIN.

In Wednesday's "Times" Mr. Maurice Baring gave a striking instance of the late Russian Premier's courage in rescuing some members of the medical profession from being lynched at the hands of the infuriated peasantry. In the course of his letter Mr. Baring said: "In 1905 agrarian riots broke out in the government of Saratov, where M. Stolypin was Governor. In the district of Balashev 2,000 men had taken the law into their own hands and had condemned the whole of the medical staff to be lynched. M. Stolypin prevented this by his own unaided personal action, and not by bullets and bayonets. He was injured in the hand, and, as it was, two doctors, Shmelev and Nevzorov, were killed. Had it not been for M. Stolypin's extraordinary courageous action, all members of the educated middle class who were there would undoubtedly have been lynched." It would seem clear at any rate that M. Stolypin, whatever his political mistakes, was a brave man. Poetical justice decided that medical assistance should come to *him* when it was too late.

### ACID-RESISTING MACKINTOSH.

The surprises of our "Question Box Column" are not only for the opener of the questions. Sometimes they strike the questioner in an unexpected way. A. C. M., for instance, who inquired for an acid-resisting mackintosh in a recent issue was probably a little overwhelmed with the information that greeted him from unexpected and interested quarters. Our correspondent did not seem to be aware that acid-resisting mackintosh has been made a special study of by a certain class of waterproof or rubber manufacturers. It is a specialty, a fabric made for a specific purpose. We commend to his notice the catalogue of Messrs. Anderson, Anderson, & Anderson, of 37 Queen Victoria Street, E.C., a firm which has devoted many years' study to the production of acid-resisting substances. As for the one person intelligent enough to ask a question there are always one hundred glad to have the answer, we make no apology for referring to this question at length.

### THIS WEEK'S DRUG MARKET.

Business has been rather better during the week, and there are signs that a further improvement will set in as the season advances. The general tendency of prices is in an upward direction. Cocaine has been in better demand and an advance in price is not altogether improbable. Citric acid continues to be in good demand and the price has advanced slightly. Opium is again dearer, as also are morphine and codeine. An advance in the price of British grain spirit is expected to be announced shortly. Saffron is tending higher in value, the drought in Spain having affected the growing plants unfavourably. Acetyl-salicylic acid is dearer, and the prices of collodion and tannic acid have been slightly advanced.

